# Life cycle studies of the hexose transporter of *Plasmodium* species and genetic validation of their essentiality

**DOI:** 10.1111/j.1365-2958.2010.07060.x

**Published:** 2010-02-04

**Authors:** Ksenija Slavic, Ursula Straschil, Luc Reininger, Christian Doerig, Christophe Morin, Rita Tewari, Sanjeev Krishna

**Affiliations:** 1 Centre for Infection, Cellular and Molecular Medicine, St. George's University of LondonLondon SW17 0RE, UK; 2 Division of Cell and Molecular Biology, Imperial College LondonLondon, UK; 3 INSERM U609 Wellcome Centre for Molecular Parasitology, University of GlasgowGlasgow, UK; 4 INSERM U609, Global Health Institute, Ecole Polytechnique Fédérale de Lausanne (EPFL)Station 19, CH-1015 Lausanne, Switzerland; 5 Département de Chimie Moléculaire (UMR 5250, ICMG FR-2607, CNRS) Université Joseph FourierGrenoble Cedex, France; 6 Institute of Genetics, School of Biology, The University of NottinghamNottingham, UK

## Abstract

A *Plasmodium falciparum*hexose transporter (PfHT) has previously been shown to be a facilitative glucose and fructose transporter. Its expression in *Xenopus laevis*oocytes and the use of a glucose analogue inhibitor permitted chemical validation of PfHT as a novel drug target. Following recent re-annotations of the *P. falciparum* genome, other putative sugar transporters have been identified. To investigate further if PfHT is the key supplier of hexose to *P. falciparum* and to extend studies to different stages of *Plasmodium* spp., we functionally analysed the hexose transporters of both the human parasite *P. falciparum* and the rodent parasite *Plasmodium berghei* using gene targeting strategies. We show here the essential function of *pfht* for the erythrocytic parasite growth as it was not possible to knockout *pfht* unless the gene was complemented by an episomal construct. Also, we show that parasites are rescued from the toxic effect of a glucose analogue inhibitor when *pfht* is overexpressed in these transfectants. We found that the rodent malaria parasite orthologue, *P. berghei* hexose transporter (PbHT) gene, was similarly refractory to knockout attempts. However, using a single cross-over transfection strategy, we generated transgenic *P. berghei* parasites expressing a PbHT–GFP fusion protein suggesting that locus is amenable for gene targeting. Analysis of *pbht-gfp* transgenic parasites showed that PbHT is constitutively expressed through all the stages in the mosquito host in addition to asexual stages. These results provide genetic support for prioritizing PfHT as a target for novel antimalarials that can inhibit glucose uptake and kill parasites, as well as unveiling the expression of this hexose transporter in mosquito stages of the parasite, where it is also likely to be critical for survival.

## Introduction

Malaria still afflicts around 500 million people and continues to kill around 1 million children a year. The recent emergence of resistance to artemisinin (Noedl *et al.*, 2008; Dondorp *et al.*, 2009), the last line of defence against multi-resistant parasites in some parts of the world, makes the search for new antimalarial drug targets an urgent one.

Although transport proteins are excellent drug targets in other systems (Imming *et al.*, 2006), they are underexploited as targets in *Plasmodium falciparum*, the most pathogenic malarial parasite, and other *Plasmodium* spp. (Cowman and Crabb, 2003).

We first identified the hexose transporter of *P. falciparum* (PfHT, PFB0210c) as a potential drug target by studying its function after expression in *Xenopus laevis*oocytes, and demonstrating selective inhibition of PfHT versus mammalian orthologues using an *o-*3 undecenyl glucose derivative (CM3361) (Joet *et al.*, 2003). These studies were extended to the *Plasmodium berghei* murine model, to establish that CM3361 can attenuate parasitaemias *in vivo*. There is also added value (in addition to parasite clearance) in inhibiting glucose uptake by infected erythrocytes, because competition for this substrate between the parasite and the host tissue may be eliminated rapidly. Interfering with metabolic diversion of glucose may be particularly important in cerebral tissues in syndromes like cerebral malaria, where glucose delivery may become rate limiting.

PfHT is a facilitative hexose transporter in the Major Facilitator Superfamily of integral membrane proteins that mediates the uptake of glucose and fructose by the parasite (Woodrow *et al.*, 1999). *Pfht* is a single copy gene in the *P. falciparum*genome with no close paralogues. Three other proteins, PFI0955w, PFI0785c and PFE1455w have been annotated with putative sugar transport function (Gardner *et al.*, 2002; Martin *et al.*, 2005), although they have diverged considerably from typical sugar transporters (21%, 13% and 7% amino acid sequence identity compared with PfHT, respectively). Unlike PfHT, PFI0955w and PFI0785c are expressed only late in the asexual cycle. Together with chemical validation data demonstrating that PfHT is essential for parasite survival, this makes it unlikely that PFI0955w and PFI0785c function in alternative mechanisms for delivery of hexoses as energy substrates for *P. falciparum*. To test this hypothesis, we performed gene transfection experiments aiming at determining the essentiality (or otherwise) of *pfht* in *P. falciparum* and of its orthologue in *P. berghei* (*pbht*, PB000562.01.0). We also generated *Pbht-gfp* transgenic parasites and employed them in the visualization of expression of this transporter during malaria parasite development. In addition, we also attempted to correlate the level of *pfht* expression in a transfected *P. falciparum* line with susceptibility to a specific inhibitor, CM3361.

## Results

### Pfht is indispensable for the erythrocytic development in *P. falciparum*

**Fig. 1:**
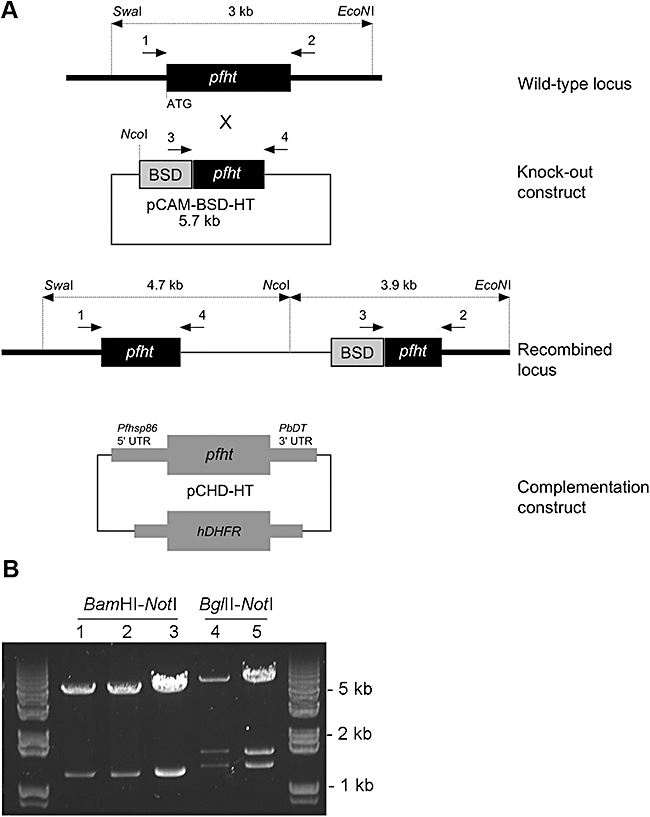
A. Strategy for disruption of the PfHT gene. Single cross-over homologous recombination of the knockout plasmid and the endogenous gene results with two truncated copies of the gene. The location of PCR primers is indicated by numbered arrows. Restriction sites of enzymes used to digest genomic DNA prior to Southern blotting and expected Southern blot fragments are also indicated. The complementation construct (pCHD-HT) allows *pfht* expression under the *Pfhsp86* promoter. B. Plasmid rescue of episomes from transfected parasites. Lane 1: re-isolated episome from parasites transfected with the knockout construct (pCAM-BSD-HT). Lanes 2 and 4: re-isolated episomes from parasites co-transfected with the knockout and the complementation construct (pCHD-HT). Lane 3: pCAM-BSD-HT plasmid. Lane 5: pCHD-HT plasmid. BamHI-NotI digestion releases a 1.2 kb knockout fragment from pCAM-BSD-HT. BglII-NotI digestion releases the PfHT gene (1.5 kb) and an additional 1.2 kb fragment from pCHD-HT.

To test if *pfht* can be disrupted during the asexual stages of *P. falciparum*, two transfection experiments were performed (Fig. 1A). In the first transfection, 3D7 parasites were electroporated with the knockout plasmid, pCAM-BSD-HT. Transfected populations were subjected to blasticidin pressure, and blasticidin-resistant parasites were readily obtained (14 days post transfection). The second transfection consisted of electroporation of 3D7 parasites with both the knockout and the complementation constructs. Double selection with blasticidin and WR99210 was applied 48 h post transfection. Doubly resistant parasites appeared in the culture somewhat later than blasticidin-resistant parasites, 19 days after the electroporation.

Episomal plasmids were recovered from all transfected parasites by plasmid rescue to confirm that parasites contained the right constructs (Fig. 1B). Bacterial clones transformed with genomic DNA from blasticidin-resistant parasites all contained pCAM-BSD-HT plasmid while bacterial clones transformed with genomic DNA from doubly resistant parasites carried either the pCAM-BSD-HT or the pCHD-HT plasmid.

Genomic DNA was isolated from both singly and doubly transfected parasites at various stages and was analysed by PCR to establish if integration of the knockout vector and disruption of *pfht* had occurred.

**Fig. 2:**
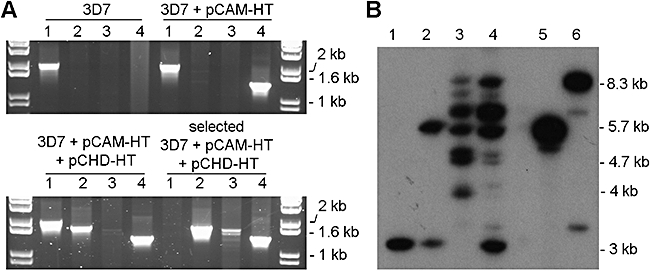
Genotype analysis of wild-type 3D7 parasites and parasites transfected with pCAM-BSD-HT alone or co-transfected with pCAM-BSD-HT and pCHD-HT. A. PCR analysis: lane 1, detection of the wild-type *pfht* locus 2 kb (primers 1 + 2, see Fig. 1); lane 2, detection of the 5′ integration of pCAM-BSD-HT into the *pfht*locus ∼1.8 kb (primers 1 + 4); lane 3, detection of the 3′ integration of pCAM-BSD-HT ∼1.7 kb (primers 3 + 2); lane 4, detection of the pCAM-BSD-HT episome 1.4 kb (primers 3 + 4). B. Southern blot analysis. Genomic DNA extracted from wild-type 3D7 parasites (lane 1), parasites transfected with pCAM-BSD-HT (lane 2), selected parasites co-transfected with pCAM-BSD-HT and pCHD-HT after blasticidin cycling – complemented parasites (lane 3) and unselected co-transfected parasites prior to blasticidin cycling (lane 4), and plasmid DNA, pCAM-BSD-HT (lane 5) and pCHD-HT (lane 6) were digested with *Swa*I, *Nco*I and *Eco*NI. The blot was probed with the 1.2 kb *pfht*fragment that was used as an insert for the pCAM-BSD-HT plasmid; wild-type locus 3 kb, integration of pCAM-BSD-HT 4.7 kb (5′) and 3.9 kb (3′), pCAM-BSD-HT episome 5.7 kb, pCHD-HT episome 8.3 kb.

We found we were not able to obtain a knockout of *pfht* in parasites transfected only with the knockout vector. PCR detected only the wild-type locus and episomal presence of the knockout construct, but did not show any amplicons diagnostic for integration of the construct into the *pfht* locus (Fig. 2A). This suggests that the *pfht* gene is essential for survival of asexual stages of *P. falciparum*. Co-transfection of 3D7 parasites with both the knockout and the complementation constructs confirms that *pfht* locus is accessible for homologous recombination. PCR analysis of these parasites detected bands corresponding to the 5′ and 3′ ends of the pCAM-BSD-HT integration event (Fig. 2A). A wild-type locus was still detectable in these parasites, indicative of a genotypically diverse population of parasites. To select for parasites with a disrupted *pfht* locus, blasticidin selective pressure was removed for 3 weeks. During this time, parasites would tend to lose the pCAM-BSD-HT episome. After 3 weeks, blasticidin was reapplied for another 3 weeks and in this time parasites with pCAM-BSD-HT integrated into their genome would be positively selected (as the PfHT protein was expressed from the complementing plasmid also present in the cells). This cycling selection was repeated twice. PCR analysis on genomic DNA isolated from parasites selected in this way (below referred to as complemented) showed complete loss of the *pfht* wild-type locus, and presence of the pCAM-BSD-HT integration and episome (Fig. 2A).

Southern blot analysis was performed to confirm genotypes (Fig. 2B). When probed with a *pfht* fragment, DNA from blasticidin-resistant single-transfected parasites yielded two bands corresponding to the wild-type locus and the knockout plasmid (3 kb and 5.7 kb, respectively), while doubly resistant parasites contained pCAM-BSD-HT integrated into the *pfht* locus (4.7 kb and 3.9 kb) and complete loss of the wild-type locus after cycling with blasticidin pressure. Additionally, complemented parasites showed more complicated Southern blot patterns, suggesting that super-integration events of both plasmids may have occurred in these parasites, as previously observed in similar instances of complementation approaches (Dorin-semblat *et al.*, 2007).

### Phenotype of *P. falciparum* transgenic parasites with an overexpression of *pfht*

Our complemented transgenic *P. falciparum* parasites do not express PfHT from the endogenous locus (which is disrupted in these parasites, see Fig. 2), but from the complementing episome, which drives the expression of PfHT from the *Pfhsp86* promoter. Complemented parasites have shown a stable phenotype as their removal from the WR99210 selective pressure for a prolonged period did not result in an impaired growth upon the restoration of the selection when compared with complemented parasites continuously cultured exposed to WR99210 (not shown). This finding suggests that parasites were not losing the complementation plasmid in the absence of the selection drug.

**Fig. 3:**
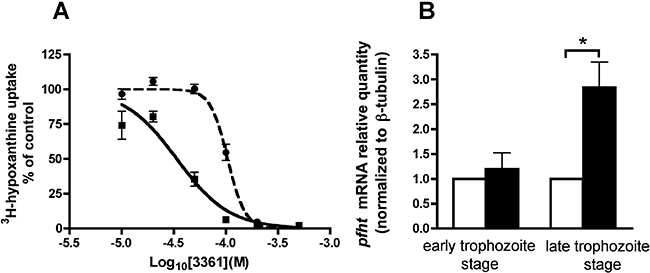
Phenotype analysis of wild-type 3D7 parasites and complemented parasites. A. Effect of compound 3361 on growth of wild-type 3D7 parasites (squares) and complemented parasites (circles). Complemented parasites have disrupted endogenous *pfht* locus, instead *pfht* is expressed from pCHD-HT episome under the *pfhsp86*promoter. Growth inhibition was measured by incorporation of ^3^H-hypoxanthine with five replicates used per inhibitor concentration. The experiment was repeated five times. Result of a single experiment is shown. Obtained 3361 IC_50_ values for 3D7 and complemented parasites were 44.2 ± 8.7 and 109.5 ± 9.6 µM respectively (*P* = 0.001 student's *t*-test, unpaired, two-tailed, *n* = 5). B. Real-time PCR analysis of *pfht*expression normalized to β*-tubulin* in wild-type 3D7 (white bars) and complemented parasites (black bars) (result of 6 experiments with 3 replicates each, one sample *t*-test, *P = 0.015).

Because the *Pfhsp86* promoter is highly active throughout the asexual cycle, it can be predicted that PfHT levels are higher in the transgenic parasites than in wild-type cells. To test this hypothesis, we measured the sensitivity of both lines to CM3361, a specific inhibitor of PfHT. When compared with wild-type 3D7, complemented parasites with episomal *pfht* expression showed a 2.5-fold increase in IC_50_ value for CM3361 (44.2 ± 8.7 and 109.5 ± 9.6 µM, respectively; *P* = 0.001 student's *t*-test, unpaired, two-tailed, *n* = 5) (Fig. 3A). The sensitivity to chloroquine was the same for these two strains of parasites (wild type: 17.6 ± 4.8 nM, *n* = 4; complemented transfectants: 15.3 ± 2.6 nM, *n* = 3; *P* = 0.7 student's *t*-test, unpaired, two-tailed). The possibility exists that the 2.5-fold shift in the parasite sensitivity to 3361 is due to an off-target effect as a result of selection with blasticidin and WR99210 although this is less likely given the parasite's unaffected sensitivity to chloroquine, an unrelated compound.

Using quantitative PCR technology, *pfht* expression was compared at ring (∼10 h post invasion) and trophozoite stages (∼30 h post invasion) in transfected and control cell lines. Significantly increased *pfht* expression at the trophozoite stage (approximately threefold) was found in complemented parasites compared with 3D7 parasites. In ring-stage parasites there was no difference between the experimental groups (Fig. 3B).

### PbHT is essential for erythrocytic stages of *P. berghei*

**Fig. 4:**
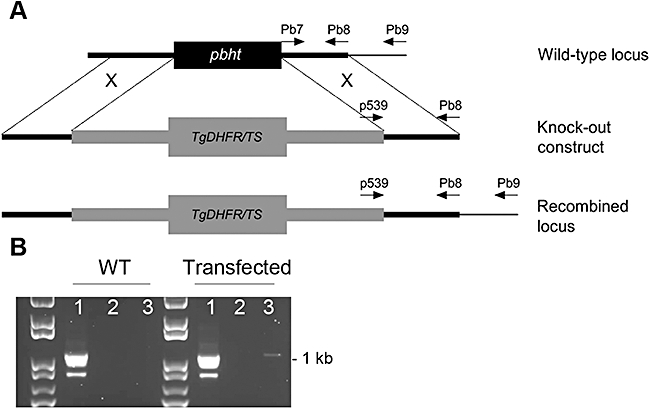
*Pbht* knockout attempt. A. A double cross-over knockout strategy used to attempt a knockout of *pbht* and investigate its function; the locations of primers used for PCR analysis of the locus are indicated by arrows. Tg DHFR/TS, *Toxoplasma gondii* dihydrofolate reductase/thymidylate synthase. B. PCR analysis of the *pbht* locus in wild type (WT) and parasites transfected with the knockout construct; lane 1, detection of the wild-type locus 1.1 kb (primers Pb7 + Pb9); lane 2, integration detection 1.2 kb (primers p539 + Pb9); lane 3, episome detection 1.1 kb (primers p539 + Pb8).

**Fig. 5:**
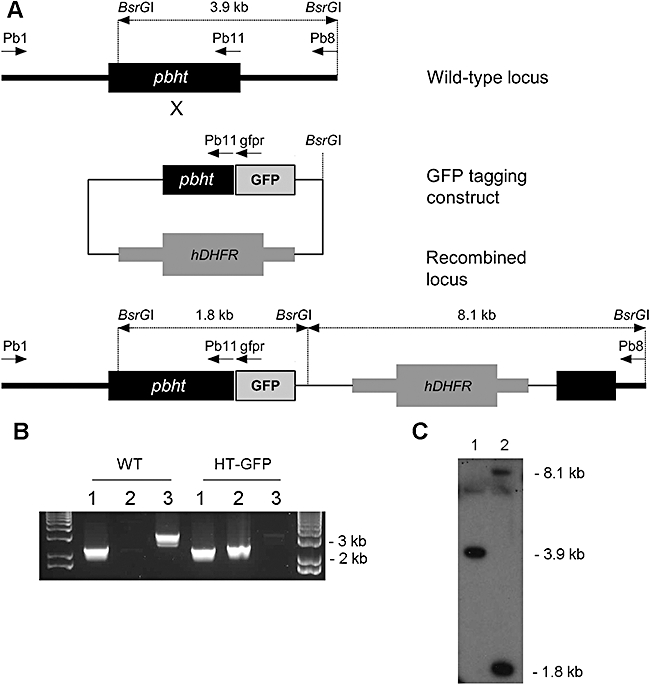
Tagging of the *pbht* locus with GFP. A. The strategy for GFP-tagging of the *pbht* locus; arrows indicate the location of PCR primers. B. PCR analysis of the wild type (WT) and a pyrimethamine-resistant *pbht-gfp* transfected line. Lane 1, positive control 2.3 kb (primers Pb1 + Pb11); lane 2, detection of the gfp-tagged locus 2.4 kb (Pb1 + gfpr); lane 3, detection of the wild-type locus 3.2 kb (Pb1 + Pb8). C. Southern blot analysis of wild-type (1) and *pbht-gfp* transfected line (2). gDNA was digested with *BsrG*I, and blot was probed with a *pbht* fragment used for generation of the tagging construct; wild-type locus 3.9 kb, integration bands 1.8 kb (5′) and 8.1 kb (3′).

Taking into account reported differences between *P. falciparum* and *P. berghei*, we were interested to investigate if the *P. berghei* hexose transporter (PbHT) is also essential for parasite growth similarly to its *P. falciparum* orthologue. *Pbht* 5′ and 3′ untranslated regions were cloned upstream and downstream of the dihydrofolate reductase (DHFR) cassette, respectively, in the knockout construct designed for double cross-over homologous recombination. Under pyrimethamine drug pressure, parasites containing the DHFR cassette were selected after three independent transfection experiments. PCR on genomic DNA obtained from all three drug-resistant populations did not detect any integration of the knockout construct or disruption of *pbht*, suggesting the essential function of the gene (Fig. 4). In contrast, we readily obtained transfected *P. berghei* parasites containing the integration of the PbHT-GFP construct into the *pbht* locus. This generated transgenic parasites expressing C-terminally GFP-tagged PbHT, which demonstrates that the locus is accessible for genetic targeting and confirms the essentiality of the hexose transporter gene for completion of the erythrocytic asexual cycle of malaria parasites (Fig. 5).

### Expression profile of *pbht-gfp* during *P. berghei* life cycle

**Fig. 6:**
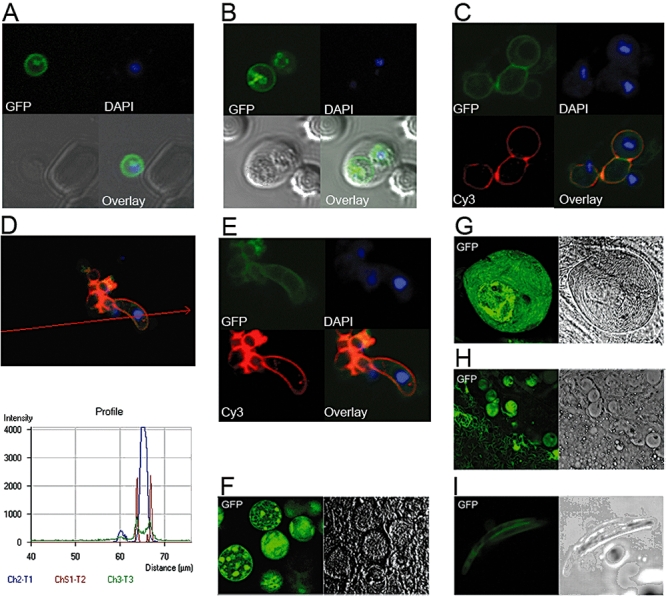
Direct fluorescence imaging of *pbht-gfp* transgenic line. A. A young, blood-stage *pbht-gfp P. berghei* parasite. B. Two *pbht-gfp P. berghei*trophozoites inside an erythrocyte. C. Zygotes/or female gametes; interestingly a smaller round cell containing surface GFP fluorescence but no P28 staining is also observed. D. Analysis of fluorescence intensities across the ookinete cell. E. Ookinete [C–E: live parasites from 20 to 24 h culture in the ookinete medium, immunostained with a monoclonal antibody against the female gamete/zygote/ookinete marker P28 (red); DAPI was used as a nuclear dye (blue)]. F. *A. stephensi* midgut 11 days post infection. G. Sporulating midgut oocyst 21 days post infection. H. Sporozoites released from ruptured midgut oocysts. I. Sporozoites released from salivary glands of *A. stephensi* 21 days post infection.

Available microarray data (PlasmoDB, Version 6.2) indicate that PfHT mRNA is detectable throughout the erythrocytic stages of development. Direct fluorescence imaging of blood stages of *pbht-gfp* transgenic parasites reveals fluorescence signal associated with the parasite's plasma membrane present in both early and late blood stages (Fig. 6A and B), demonstrating that PbHT protein is indeed present throughout the asexual cycle. Some of the observed fluorescence signal is internal and may be associated with perinuclear localization as well as the food vacuole, although colocalization studies are needed to ascertain this point. Observed internal PbHT-GFP signal could be associated with developing PbHT in its trafficking pathway or may come from a small proportion of PbHT-GFP protein that has been mislocalized. It is noteworthy that taken together with all our other work supporting the essentiality of this transporter, mislocalization of a greater proportion of the protein would have had functional consequences that have not been observed.

Very limited information is available regarding glucose uptake during the sexual stages of malaria parasite development especially during its development within the mosquito. We used the *pbht-gfp* transgenic parasites to investigate the expression of this transporter during the parasite's development in the mosquito host.

We analysed PbHT-GFP expression in live parasites from an ookinete-enriched culture, and showed its expression and colocalization with P28, a surface marker of the ookinete, female gamete and zygote (Fig. 6C–E).

Analysis of mosquito midgut oocysts dissected 11 days post infection showed internal foci of fluorescence of developing sporoblasts and lower intensity membrane fluorescence signal (Fig. 6F). Twenty-one days post infection, strong fluorescence signal was detectable from sporulating oocysts and oocyst-derived sporozoites (Fig. 6G and H). Similarly, sporozoites released from dissected salivary glands 21 days post infection showed expression of PbHT-GFP (Fig. 6I).

**Fig. 7:**
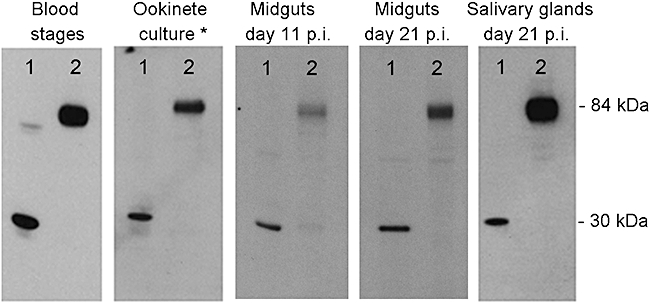
Western blot analysis. Ten micrograms of parasite material from asexual blood stages, ookinete-enriched culture and mosquito midguts and salivary glands stages of PbGFP_CON_ (lane 1) and *pbht-gfp* line (lane 2) was subjected to SDS-PAGE (10% acrylamide), transferred to a nitrocellulose membrane and probed with 1:1000 diluted anti-GFP antibody (Roche) and 1:3000 diluted HRP-conjugated anti-mouse antibody. *Ookinete-enriched culture obtained by incubation of blood sample of an infected mouse in the ookinete medium overnight at 19°C. p.i., post infection.

Expression of PbHT-GFP in parasites from all analysed developmental stages was confirmed by Western blotting using an anti-GFP monoclonal antibody (Fig. 7). As a positive control for blotting and antibody staining, we used PbGFP_CON_ line, a *P. berghei* line that constitutively expresses GFP throughout the life cycle (Franke-Fayard *et al.*, 2004). A protein band of ∼82 kDa appeared for all analysed *pbht-gfp* samples, which corresponds to a predicted mass of PbHT–GFP fusion protein (84.4 kDa).

## Discussion

Identifying inhibitors of the parasite's essential nutrient uptake pathways may lead to the development of novel antimalarials. The *P. falciparum* hexose transporter, PfHT, has previously been investigated using chemical validation methodologies, which suggested that a functional PfHT molecule is required for parasite survival (Joet *et al.*, 2003). The present study now provides genetic evidence that PfHT is indeed indispensable for erythrocytic stages of *P. falciparum*. *Pfht* could not be disrupted in the genome unless its expression was maintained by an exogenously supplied construct. We have further predicted that altered *pfht* expression would lead to changes in the parasite's sensitivity to a specific inhibitor of PfHT, 3361. We therefore investigated the transfected line containing PfHT expressed only from a complementing episome and lacking endogenous *pfht*. Quantitative PCR showed significantly increased expression of *pfht* in these parasites compared with wild-type controls at the trophozoite stage, whereas expression at ring stages was not affected. A previous study showed the peak of *pfht* expression to be at the early ring stage (8 h post invasion), with falling expression at the late ring stage (16 h) and intermediate levels of expression at the trophozoite stage (Woodrow *et al.*, 1999). This indicates that highly regulated *pfht* expression in wild-type parasites can be replaced by higher but less regulated *pfht* expression in transfected lines. In support of this finding, the transfected line was also more resistant to growth inhibition by CM3361 (2.5-fold increase in mean IC_50_ values compared with controls; *P* < 0.001). These findings further validate PfHT as a novel target and provide strong evidence that PfHT is the specific target of CM3361 in parasites.

Although there is a growing evidence for a functional TCA cycle in blood stages of *P. falciparum*, its role is not fully defined. Expression profiling studies of *P. falciparum* parasites from patients found three metabolically distinct clusters: a glycolytic metabolism state, a starvation response with induction of metabolism of alternative carbon sources and an environmental stress response (Daily *et al.*, 2007). The study by Daily *et al.* suggests that parasites can undergo a metabolic shift *in vivo*, switching from predominately glycolytic metabolism to metabolism of alternative carbon sources with induction of gene sets associated with function of mitochondria and apicoplast. Despite the complexity of metabolic patterning that emerges from these types of studies, glucose delivery is nevertheless essential for parasite survival and may also be critical for metabolic diversion of this key substrate from host tissues, thereby exacerbating disease processes. Here, when taken together with previous studies on PfHT, the central role of PfHT in hexose delivery to parasites is confirmed.

We have also extended our analysis of the *Plasmodium* hexose transporter to a rodent malaria model, *P. berghei*, which enabled studies with insect stages of the parasite's life cycle. Energy requirements and metabolism during parasite development in the invertebrate host are largely unknown, although it is established that gametocytes display increased expression of TCA cycle and oxidative phosphorylation enzymes compared with asexual stages (Hall *et al.*, 2005). In addition to asexual blood stages, proteome analyses of gametocytes, ookinetes, mosquito midgut oocysts and sporozoites have been performed. Proteomic survey of *P. berghei* life cycle has detected PbHT expression in asexual blood stages (Hall *et al.*, 2005) and gametocytes (Khan *et al.*, 2005). Similarly, *P. falciparum* proteome studies identified PfHT expression in asexual stages and gametocytes (Florens *et al.*, 2002; Lasonder *et al.*, 2002). More recently, PfHT has been found expressed in oocyst-derived sporozoites (Lasonder *et al.*, 2008). These findings may reflect in part methodological limitations of applying current proteomic approaches to the detection of integral membrane proteins. They suggest that other membrane proteins should also be examined individually for expression in different stages of the life cycle. Here we show, by direct fluorescence as well as Western blot analysis, that PbHT is expressed in female gametes/or zygotes, ookinetes, midgut oocysts and sporozoites derived from both midgut oocysts and salivary glands.

Furthermore, the importance of glucose transport for a cell may be inferred from the presence of expressed glycolytic enzymes. In the *P. falciparum* sporozoite proteome, with an exception of 6-phosphofructokinase, all glycolytic enzymes have been detected. However, this enzyme has been detected in the *P. berghei* sporozoite proteome, indicating that its absence from *P. falciparum* sporozoite proteome is probably due to the limitations of detection technology rather than true absence. Therefore, available proteomic data and our data presented here together suggest that the sugar uptake and glycolysis are present in sporozoites. Joet *et al.* (2002) analysed temperature dependence of *Plasmodium* hexose transporters activity and showed that they are active in a broad temperature range, with approximately 50 % of maximal activity retained at 20°C. This finding shows that these transporters can function in insect stages of development that take place at 22–26°C.

Further studies will evaluate potential of specific PfHT inhibitors in reducing gametocytaemia in culture. Novel drugs that would have activity against both asexual blood stages and gametocytes are necessary if the malaria research community is aiming towards the eradication of the disease.

These studies may help in identifying inhibitors of PfHT that can contribute to the challenging drug discovery process. Indeed, glucose is an essential energy substrate, not only for malaria parasites, but for other unrelated parasites as well, such as the kinetoplastida (Barrett *et al.*, 1998; Burchmore *et al.*, 2003).

## Experimental procedures

### Ethics statement

All animal work has passed an ethical review process and was approved by the United Kingdom Home Office. Work was carried out in accordance with the United Kingdom ‘Animals (Scientific Procedures) Act 1986’ and in compliance with ‘European Directive 86/609/EEC’ for the protection of animals used for experimental purposes.

### Construction of plasmids for transfection of *P. falciparum*

#### pCAM-BSD-HT

A 1.2 kb fragment of the central region of *pfht*[nucleotides 163–1348 of the *pfht* open reading frame (ORF)] was amplified using primers KOHTf and KOHTr (Table 1). An amplified fragment was inserted into the pCAM-BSD plasmid using BamHI and NotI restriction sites (Sidhu *et al.*, 2005). The resulting plasmid, pCAM-BSD-HT was used in transfections as a gene disruption (knockout) vector.

**Table 1:** *P. falciparum* primer sequences.

Primer		Sequence
Knockout fragment	KOHTf	5′-CAGGATCCGTTGTAGAATTTGAATGGTGTAAAGG
	KOHTr	5′-caGCGGCCGCTAATAATGTCTGATGGGAAGACAAC
Complementation gene	XKBNf	5′-CCAAGTCGGTTGTATGAGCGGCCGCTTACCACTAAACCAGCC
	XKBNr	5′-GGCTGGTTTAGTGGTAAGCGGCCGCTCATACAACCGACTTGG
PCR detection of knockout plasmid integration	1	5′-TATATATAAAATGAGGAATTGGAAAATTTTTC
	2	5′-AATGGAATAAATGTCGATTGGATAATGTTTG
	3	5′-TATTCCTAATCATGTAAATCTTAAA
	4	5′-CAATTAACCCTCACTAAAG
Real-time PCR analysis	PfHT-f	5′-GAAATGTTTCCATCAGAAATAAAAG
	PfHT-r	5′-GAATCGAAGGGGATTTCTTAATA
	PfHT probe	5′-CAACAATAATTGCACAAACCCAATTAACTAATGA
	Tubulin-f	5′-TGATGTGCGCAAGTGATCC
	Tubulin-r	5′-TCCTTTGTGGACATTCCTTCCTC
	Tubulin probe	5′-TAGCACATGCCGTTAAATATCTTCCATGTCT

#### pCHD-HT

Xkb.1 plasmid contains the full-length *pfht* gene flanked by BglII sites and was used as a source of the *pfht* gene (Woodrow *et al.*, 1999). First, a pair of complementary PCR primers (XKBNf and XKBNr, Table 1) was used in site-directed mutagenesis to convert a 3′ BglII site immediately after the stop codon into a NotI site generating the XkbN plasmid. Subsequently, the *pfht*gene was subcloned from XkbN plasmid into a pHGB vector downstream of the *pfhsp86* promoter using BglII and NotI restriction sites (Tonkin *et al.*, 2004). The resulting plasmid, pHGB-HT, was used as an entry vector in a LR recombination reaction with the destination vector, pCHD that contains DHFR cassette conferring resistance to WR99210 (Tonkin *et al.*, 2004). After the recombination reaction, the resulting vector, pCHD-HT, contains *pfht* gene under the control of *pfhsp86* promoter and the DHFR resistance cassette, and was used in co-transfections as a complementation plasmid.

All transfection constructs were verified by sequence analysis.

### *P. falciparum* culture and transfection

*Plasmodium falciparum* 3D7 clone was grown as described previously (Trager and Jensen, 1976) and used for transfections. Parasites were sorbitol-synchronized, and ring-stage parasites were subjected to electroporation in the presence of 100 µg of pCAM-BSD-HT plasmid or 50 µg of both pCAM-BSD-HT and pCHD-HT plasmids, as described previously (Dorin-semblat *et al.*, 2007). Blasticidin (2.5 µg ml^−1^) and WR99210 (5 nM) were added 48 h after transfection to select for transformed parasites. Blasticidin-resistant parasites appeared in culture 14 days post transfection and parasites resistant to both blasticidin and WR99210 appeared 19 days after transfection.

After the detection of homologous integration of the knockout vector in doubly transfected parasites by PCR, they were subjected to blasticidin cycling to select for parasites with pCAM-BSD-HT integrated into the genome. The cycling consisted of 3 weeks of culturing without blasticidin, followed by 3 weeks with blasticidin pressure. This selection cycle was repeated twice. No decrease in parasiteamia was observed after reapplying the drug pressure.

### Genotype characterization

#### Plasmid rescue of episomes

Parasite pellets were obtained by saponin lysis and genomic DNA was extracted by proteinase K (0.1 mg ml^−1^) and SDS (2%) treatment for 2 h at 55°C. Genomic DNA extracted from both knockout and co-tranfected parasites was used to transform *E. coli*supercompetent cells. Plasmid DNA was isolated from obtained bacterial clones using standard methods and digested with *Bam*HI and *Not*I or *Bgl*II and *Not*I.

#### PCR

PCR analysis of transfectants was performed on genomic DNA using *Taq* polymerase (Sigma) and following cycling conditions: 1 cycle at 92°C (2 min); 35 cycles at 92°C (30 s), 54°C (45 s) and 65°C (1.5 min) and 1 cycle at 65°C (5 min). Sequences of primers used are shown in Table 1. PCR primers 1 and 2 anneal to the *pfht* wild-type locus outside the ORF region inserted in pCHD-HT. Primers 3 and 4 anneal to the pCAM-BSD-HT and flank the insertion site.

Four different combinations of primers were used in order to detect: the wild-type *pfht* locus (primers 1 and 2), 5′ end of integration event (primers 1 and 4), 3′ end of the integration event (primers 2 and 3) and the presence of episome (primers 3 and 4).

#### Southern blotting

Genomic DNA extracted from parasite pellet obtained by saponin lysis was digested with *Swa*I, *Nco*I and *Eco*NI. pCAM-BSD-HT and pCHD-HT plasmid DNA were also digested with same enzymes and used as controls. One microgram of digested genomic DNA and 1.5 ng of digested plasmid DNA were separated on 0.8% agarose gel and transferred to positively charged nylon membrane (Hybond-XL, Amersham). Subsequently, the membrane was hybridized overnight at 58°C with 1 kb *pfht* knockout fragment labelled with ^32^P-dCTP (Sambrook *et al.*, 1989).

### Phenotype characterization

#### Quantitative PCR

Parasites were sorbitol-synchronized and frozen down in RNA-*Later*solution (Ambion) for RNA extraction at rings (∼10 h post invasion) and trophozoite stages (∼30 h post invasion). RNA was isolated with RNA isolation kit (Qiagen) according to manufacturer's instructions. Isolated RNA was treated with DNase I (Promega) and used in reverse transcription reactions (High Capacity cDNA Reverse Transcription Kit with RNase inhibitor, Applied Biosystems).

Quantification of the gene expression was assessed by TaqMan real-time PCR (Stratagene Mx3005P QPCR System). Primers and probes are summarized in Table 1. The *pfht* probe was FAM-labelled at the 5′ end, and the β-*tubulin* probe was VIC-labelled. Both probes had a TAMRA quencher at the 3′ end. Amplification reactions were done as duplex PCR in 96-well plates using TaqMan Universal PCR Master Mix (Applied Biosystems). *pfht*primers and probe concentrations were 600 nM and 40 nM respectively. β*-tubulin* primers and probe were all used at 100 nM (Price *et al.*, 2004). The following cycling conditions were applied: 95°C 10 min, followed by 45 cycles of 95°C (15 s) and 57°C (1 min). Reactions were done in triplicate and experiments repeated three times.

Relative *pfht* expression in complemented parasites compared with calibrator (3D7 parasites) was calculated using the efficiency-corrected comparative quantification method. β-*tubulin* was used as a normalizer gene. A standard curve was run together with experimental samples during each run to estimate the efficiencies of target (*pfht*) and normalizer (β-*tubulin*) assays. The following equation was used to calculate relative quantity to the calibrator: (1 + *E*_GOI_)^ΔCtGOI^/(1 + *E*_norm_)^ΔCtnorm^, where *E*_GOI_ and *E*_norm_ are efficiencies of the target and the normalizer assays and ΔCt = (Ct_calibrator_ – Ct_unknown_). Primers sequences are in Table 1.

#### Parasite growth and sensitivity to CM3361

3-O-(undec-10-en)-yl-D-glucose (CM3361) was synthesized as described earlier (Ikekawa *et al.*, 1987). The effect of glucose analogue inhibitor CM3361 on the growth inhibition of the wild-type and complemented parasites was determined using the [^3^H]-hypoxanthine incorporation assay as previously described. Ring-stage parasites were seeded in the 96-well plates at 0.5% parasitemia and 2% hematocrit in the presence of increasing concentrations of the inhibitor (five replicates per inhibitor concentration). [^3^H]-hypoxanthine was added after 24 h of incubation and parasites harvested after 48 h. Five independent experiments were performed.

#### Statistical analysis

For *P. falciparum* IC_50_ assays, a sigmoidal dose–response model with the variable slope was fitted to results using GraphPad Prism (Version 4 for Macintosh). IC_50_ values obtained with the wild-type and the complemented parasite line were compared using Student's *t*-test (unpaired, two-tailed). For the analysis of *pfht* mRNA expression, the relative *pfht* expression in the complemented parasite line was compared with 3D7 parasites using one sample *t*-test. Statistical significance was set at *P* ≤ 0.05.

### *P. berghei* culture and transfection

Transfection experiments were performed on *P. berghei* ANKA strain 2.34 parasites according to a described protocol (Janse *et al.*, 2006). The *pbht* knockout vectors were constructed for a double cross-over homologous recombination in the pBS-DHFR plasmid that contains a *Toxoplasma gondii dhfr/ts* cassette conferring resistance to pyrimethamine (Dessens *et al.*, 1999). The knockout construct was generated by inserting 0.8 kb of the *pbht* 5′ untranslated (UTR) region upstream and 1 kb of the *pbht* 3′ UTR region downstream of the *dhfr* cassette (sequences of primers used to amplify fragments from *P. berghei* genomic DNA are given in Table 2). The final knockout construct was digested with KpnI and NotI to release the fragment for transfection.

**Table 2:** *P. berghei* primers sequences.

Primer		Sequence
PbHT 5′ UTR	Pb1	5′-cgaGGTACCGTGTAAAAATTTATCGTTAAGAGAG
	Pb2	5′-CATGGGCCCTTTTTTCGTATTAATACACATATATTTCTTG
PfHT ORF	Pb3	5′-AAAGGGCCCATGACGAAAAGTTCGAAAGAT
	Pb4	5′-caaGTCGACTCATACAACCGACTTGGTCATATG
Spacer 3′ UTR	Pb5	5′-atcGTCGACATTTTGATACGCATAAATCGTATAG
	Pb6	5′-catAAGCTTTTTGATACATATATATTTGATACATATATATTTG
PbHT 3′ UTR	Pb7	5′-gtcGATATCATTTTGATACGCATAAATCGTATAGATATAG
	Pb8	5′-caaGCGGCCGCAAAAAAATAGAAATCAAATGATATATATTTACCC
PbHT-GFP fragment	Pb10	5′-catGGTACCTAACATTTGGAATATTTGTTGCAGTTTTATTGGG
	Pb11	5′-caaGGGCCCAACTCTTGATTTGCTTATATGTTTTTGTCTTTCTTC
Integration detection	Pb9	5′-CACCATTTTATTCACCATATTTTTAC
	539	5′-CAATGATTCATAAATAGTTGGACTTG
	gfpr	5′-ACGCTGAACTTGTGGCCG

To generate a *pbht-gfp* construct for a single cross-over homologous recombination, a 1 kb region of the *pbht* without the stop codon was inserted in frame and upstream of the *gfp* sequence in the plasmid p277 containing the human *dhfr* cassette and conveying resistance to pyrimethamine (Liu *et al.*, 2008). Prior to transfection, the final construct was digested with BstXI that cuts the plasmid in the middle of the insert, which is optimal for the homologous recombination event.

Transfected *P. berghei* parasites were selected with pyrimethamine selection pressure according to a described protocol (Janse *et al.*, 2006). Transfection of *P. berghei* parasites with the knockout construct was carried out minimum of three times.

### Genotype analysis of *P. berghei* transfectants

#### PCR

PCR analysis was used to inspect if the transfection construct was integrated into the correct locus in pyrimethamine-resistant parasites. For the analysis of parasites transfected with the *pbht* knockout construct, combinations of the following four primers were used to explore the *pbht* locus: primer 539 anneals to the DHFR cassette in the construct, primers Pb7 and Pb8 anneal to the *pbht* 3′ UTR inserted in the construct, whereas primer Pb9 anneals further downstream from Pb8 in *pbht* 3′ UTR and does not anneal to the sequences present in the construct. For the analysis of *pbht-GFP* transgenic parasites, Pb1, Pb8, Pb11 and gfpr primers were used.

#### Southern blot

Genomic DNA extracted from *pbht-GFP* parasite pellet obtained by lysis with NH_4_Cl was digested with *BsrG*I. One microgram of digested genomic DNA was separated on 0.8% agarose gel and transferred to a positively charged nylon membrane (Hybond-XL, Amersham) that was hybridized overnight at 55°C with 1 kb *pbht* probe labelled with ^32^P-dCTP.

### Analysis of the HT- GFP localization during malaria life cycle

Images of GFP-expressing parasites were captured with LSM 510 META Confocal Laser Scanning Microscope (Zeiss) and Leica SP5 confocal microscope. Hoechst33342 or DAPI were used for nuclear staining of blood stages parasites and ookinetes. *Anopheles stephensi* mosquitoes were fed on mice infected with *P. berghei pbht-gfp* and PbGFP_CON_ parasites as described (Sinden *et al.*, 2002). Eleven days post infection, mosquitoes were dissected and midguts were collected for direct fluorescence imaging and Western blotting analysis (∼30 midguts per parasite strain). Dissection of mosquitoes was repeated at day 21 post infection when midguts and salivary glands were collected for the analysis of *pbht* expression.

#### Western blot analysis

For Western blot analysis parasite pellets of *pbht*-GFP and PbGFP_CON_ (Franke-Fayard *et al.*, 2004). *P. berghei* blood stages were obtained by erythrocytes lysis with NH_4_Cl. Pellets of mosquitoes' midguts and salivary glands were ground with pestle and pellets resuspended in PBS containing Complete™ protease inhibitor cocktail tablets (Roche). Approximately 10 µg of each extract were heated at 70°C in NuPAGE® LDS Sample Buffer (Invitrogen), separated on Novex® 10% Bis-Tris gel (Invitrogen) and electro-transferred to a nitrocellulose membrane (Amersham). Membranes were blocked overnight at 4°C in PBS containing 5% skimmed milk and 0.1% Tween 20. Blots were probed with a mouse monoclonal GFP antibody (Roche), diluted 1:1000. Bound antibodies were detected with a HRP-conjugated anti-mouse secondary antibody, diluted 1:3000 (Bio-Rad) and ECL Western Blotting detection reagents (Amersham).

#### Ookinete culture

Blood was taken from a *pbht-gfp* infected mouse on day 4 post infection into a heparinized syringe, mixed with the ookinete culture medium (RPMI1640 containing 25 mM HEPES, 25% fetal bovine serum, 10 mM sodium bicarbonate, 50 µM xanthurenic acid, pH 7.6) and cultured at 19°C for a further 21–24 h. For direct immunolabelling cultured cells were pelleted for 2 min at 800 g and then labelled for 10 min on ice in 50 µl of ookinete medium containing Hoechst33342 and Cy3-conjugated mouse monoclonal antibody specific for P28 (Reininger *et al.*, 2009).

## References

[b1] Barrett MP, Tetaud E, Seyfang A, Bringaud F, Baltz T (1998). *Trypanosome*glucose transporters. Mol Biochem Parasitol.

[b2] Burchmore RJ, Rodriguez-Contreras D, McBride K, Merkel P, Barrett MP, Modi G (2003). Genetic characterization of glucose transporter function in *Leishmania mexicana*. Proc Natl Acad Sci USA.

[b3] Cowman AF, Crabb BS (2003). Functional genomics: identifying drug targets for parasitic diseases. Trends Parasitol.

[b4] Daily JP, Scanfeld D, Pochet N, Le Roch K, Plouffe D, Kamal M (2007). Distinct physiological states of *Plasmodium falciparum* in malaria-infected patients. Nature.

[b5] Dessens JT, Beetsma AL, Dimopoulos G, Wengelnik K, Crisanti A, Kafatos FC, Sinden RE (1999). CTRP is essential for mosquito infection by malaria ookinetes. EMBO J.

[b6] Dondorp AM, Nosten F, Yi P, Das D, Phyo AP, Tarning J (2009). Artemisinin resistance in *Plasmodium falciparum* malaria. N Engl J Med.

[b7] Dorin-Semblat D, Quashie N, Halbert J, Sicard A, Doerig C, Peat E (2007). Functional characterization of both MAP kinases of the human malaria parasite *Plasmodium falciparum* by reverse genetics. Mol Microbiol.

[b8] Florens L, Washburn MP, Raine JD, Anthony RM, Grainger M, Haynes JD (2002). A proteomic view of the *Plasmodium falciparum* life cycle. Nature.

[b9] Franke-Fayard B, Trueman H, Ramesar J, Mendoza J, van der Keur M, van der Linden R (2004). A *Plasmodium berghei* reference line that constitutively expresses GFP at a high level throughout the complete life cycle. Mol Biochem Parasitol.

[b10] Gardner MJ, Hall N, Fung E, White O, Berriman M, Hyman RW (2002). Genome sequence of the human malaria parasite *Plasmodium falciparum*. Nature.

[b11] Hall N, Karras M, Raine JD, Carlton JM, Kooij TW, Berriman M (2005). A comprehensive survey of the *Plasmodium* life cycle by genomic, transcriptomic, and proteomic analyses. Science.

[b12] Ikekawa T, Irinoda K, Saze K, Katori T, Matsuda H, Ohkawa M, Kosik M (1987). Studies on synthesis of 3-O-alkyl-D-glucose and 3-O-alkyl-D-allose derivatives and their biological activities. Chem Pharm Bull (Tokyo).

[b13] Imming P, Sinning C, Meyer A (2006). Drugs, their targets and the nature and number of drug targets. Nat Rev Drug Discov.

[b14] Janse CJ, Ramesar J, Waters AP (2006). High-efficiency transfection and drug selection of genetically transformed blood stages of the rodent malaria parasite *Plasmodium berghei*. Nat Protoc.

[b15] Joet T, Holterman L, Stedman TT, Kocken CH, Van Der Wel A, Thomas AW, Krishna S (2002). Comparative characterization of hexose transporters of *Plasmodium knowlesi, Plasmodium yoelii*and *Toxoplasma gondii* highlights functional differences within the apicomplexan family. Biochem J.

[b16] Joet T, Eckstein-Ludwig U, Morin C, Krishna S (2003). Validation of the hexose transporter of *Plasmodium falciparum* as a novel drug target. Proc Natl Acad Sci USA.

[b17] Khan SM, Franke-Fayard B, Mair GR, Lasonder E, Janse CJ, Mann M, Waters AP (2005). Proteome analysis of separated male and female gametocytes reveals novel sex-specific *Plasmodium* biology. Cell.

[b18] Lasonder E, Ishihama Y, Andersen JS, Vermunt AM, Pain A, Sauerwein RW (2002). Analysis of the *Plasmodium falciparum* proteome by high-accuracy mass spectrometry. Nature.

[b19] Lasonder E, Janse CJ, van Gemert GJ, Mair GR, Vermunt AM, Douradinha BG (2008). Proteomic profiling of *Plasmodium* sporozoite maturation identifies new proteins essential for parasite development and infectivity. PLoS Pathog.

[b20] Liu Y, Tewari R, Ning J, Blagborough AM, Garbom S, Pei J (2008). The conserved plant sterility gene HAP2 functions after attachment of fusogenic membranes in *Chlamydomonas*and *Plasmodium* gametes. Genes Dev.

[b21] Martin RE, Henry RI, Abbey JL, Clements JD, Kirk K (2005). The ‘permeome’ of the malaria parasite: an overview of the membrane transport proteins of *Plasmodium falciparum*. Genome Biol.

[b22] Noedl H, Se Y, Schaecher K, Smith BL, Socheat D, Fukuda MM (2008). Evidence of artemisinin-resistant malaria in western Cambodia. N Engl J Med.

[b23] Price RN, Uhlemann AC, Brockman A, McGready R, Ashley E, Phaipun L (2004). Mefloquine resistance in *Plasmodium falciparum* and increased pfmdr1 gene copy number. Lancet.

[b24] Reininger L, Tewari R, Fennell C, Holland Z, Goldring D, Ranford-Cartwright L (2009). An essential role for the *Plasmodium* Nek-2 Nima-related protein kinase in the sexual development of malaria parasites. J Biol Chem.

[b25] Sambrook J, Fritsch EF, Maniatis T (1989). Molecular Cloning – A Laboratory Manual.

[b26] Sidhu AB, Valderramos SG, Fidock DA (2005). pfmdr1 mutations contribute to quinine resistance and enhance mefloquine and artemisinin sensitivity in *Plasmodium falciparum*. Mol Microbiol.

[b27] Sinden RE, Butcher G, Beetsma A, Doolan DL (2002). Maintance of the *Plasmodium berghei* life cycle. Malaria Methods and Protocols.

[b28] Tonkin CJ, van Dooren GG, Spurck TP, Struck NS, Good RT, Handman E (2004). Localization of organellar proteins in *Plasmodium falciparum* using a novel set of transfection vectors and a new immunofluorescence fixation method. Mol Biochem Parasitol.

[b29] Trager W, Jensen JB (1976). Human malaria parasites in continuous culture. Science.

[b30] Woodrow CJ, Penny JI, Krishna S (1999). Intraerythrocytic *Plasmodium falciparum* expresses a high affinity facilitative hexose transporter. J Biol Chem.

